# Bisphenol A exacerbates dry eye disease via the CASP1/GSDMD-mediated pyroptotic axis: network toxicology, mendelian randomization, and experimental validation

**DOI:** 10.3389/fphar.2026.1880522

**Published:** 2026-08-11

**Authors:** Yingchen Wang, Chenhong Jiang, Yu Zhang, Kangrui Liu, Kang Lu, Youhai Wang, Minglu Xu, Qing Wang

**Affiliations:** 1 Department of Ophthalmology, The Affiliated Hospital of Qingdao University, Qingdao, Shandong, China; 2 Qingdao Medical College, Qingdao University, Qingdao, Shandong, China; 3 Department of Ophthalmology, No.971 Hospital of the People’s Liberation Army Navy, Qingdao, Shandong, China

**Keywords:** bisphenol A, dry eye disease, mendelian randomization, molecular docking, network toxicology

## Abstract

**Background:**

Bisphenol A (BPA) is a pervasive environmental contaminant associated with various systemic toxicities. However, its specific mechanistic involvement in dry eye disease (DED) pathogenesis is not defined. This study aimed to evaluate the toxicological mechanisms of BPA in DED pathogenesis and identify the key molecular mediators driving ocular surface injury.

**Methods:**

Toxicity profiles of BPA were predicted using the ProTox and ADMETlab platforms. Network toxicology and machine learning were used to explore pathogenic pathways and molecular mechanisms. Mendelian randomization (MR) analysis was performed to assess the causal effects of candidate targets on DED. Immune infiltration analysis and Gene Set Enrichment Analysis (GSEA) were used to characterize functional features and immune associations. Molecular docking and molecular dynamics (MD) simulations evaluated the spatial engagement and stability between BPA and the hub target. Finally, *in vitro* assays (CCK-8, LDH release, propidium iodide staining, ROS detection, qRT-PCR, and Western blot) using human corneal epithelial cells (HCECs) were conducted to validate the BPA-induced cytotoxicity and the molecular mechanisms.

**Results:**

Toxicity assessments predicted significant ocular irritant and corrosive properties for BPA. Machine learning algorithms identified *CASP1* as the hub gene. MR analysis provided genetic evidence that elevated *CASP1* expression causally increases DED risk (OR = 1.11, 95% CI: 1.06–1.16). Molecular docking demonstrated stable binding affinity (−5.1 kcal/mol) between BPA and the CASP1 protein. 100-ns molecular dynamics simulations confirmed its structural equilibrium and spontaneous thermodynamic stability with an MM-PBSA binding free energy of −12.19 kcal/mol. *In vitro*, BPA exposure decreased HCEC viability, compromised membrane integrity, and triggered significant intracellular ROS accumulation. BPA significantly upregulated the mRNA and protein expression of *caspase-1*, *GSDMD*, *IL-1β*, and *IL-18*. The application of a *CASP1* inhibitor reversed these alterations and mitigated ROS accumulation.

**Conclusion:**

This study suggests that *CASP1* is a central molecular component in DED. BPA promotes ocular surface injury by activating the *CASP1*/*GSDMD*-mediated pyroptotic axis and its associated inflammatory cascade. Inhibition of *CASP1* effectively abrogates this process, suggesting potential avenues for clinical intervention.

## 1 Introduction

Bisphenol A (BPA) is an industrial chemical produced in large quantities and widely employed for the manufacture of epoxy resins and polycarbonate plastics (Xing et al., 2022; Tuzimski et al., 2019). Its extensive use in specialized paper products, medical materials, beverage and food packaging (Celar Sturm and Virant-Klun, 2023), and various consumer products has led to persistent and unavoidable human exposure (Guimarães et al., 2023; Harley et al., 2013). With global plastic production approaching 400 million tons per year, BPA contamination has become a growing concern in environmental and public health research. Exposure occurs primarily through ingestion and skin contact, allowing BPA to enter systemic circulation. As a representative endocrine-disrupting chemical, BPA can interfere with estrogen receptor–mediated signaling by exhibiting estrogenic activity, thereby perturbing endocrine regulation (Adamovsky et al., 2024). Evidence from experimental and epidemiological studies indicates that BPA exposure is linked to adverse health effects, including metabolic disorders, reproductive dysfunction, neurodevelopmental impairment, cardiovascular disease, and immune dysregulation (Costa and Cairrao, 2024; Hyun and Ka, 2024; Acconcia et al., 2015; Liu et al., 2022; Chen J. et al., 2025). Despite extensive investigation of its systemic toxicity, the effects of BPA on ocular tissues remain insufficiently characterized. In particular, its potential involvement in eye disease pathogenesis has received limited attention.

Dry eye disease (DED) is a disorder of the ocular surface characterized by a complex, multifactorial pathogenesis, with global prevalence estimates reported between 5% and 50%, with particularly elevated prevalence observed among Asian populations (Clayton, 2018). The principal hallmark of this ocular disorder is compromised tear film homeostasis, manifested by unstable tear dynamics and hyperosmolarity (Han et al., 2023), which drive a self-perpetuating pathogenic cycle (Rhee and Mah, 2017). Chronic inflammation of ocular surface, oxidative stress, neurosensory dysfunction, and epithelial barrier impairment are recognized as central mechanisms underlying DED (15). These pathological alterations lead to common clinical manifestations such as visual instability, ocular irritation and pain, resulting in substantial impairment of daily functioning and quality of life (Zemanová, 2021). Established risk factors include aging, female sex, genetic predisposition, and hormonal imbalance, highlighting the complexity of DED etiology (Britten-Jones et al., 2024; Qian and Wei, 2022). Increasing evidence indicates that environmental exposures act as external modifiers of ocular surface health and may interact with intrinsic molecular pathways to promote or exacerbate DED (Lin et al., 2022; Alves et al., 2023; Rauchman et al., 2023).

Recent evidence shows that BPA exposure disrupts immune homeostasis and activates inflammatory signaling pathways, processes that are integral to DED pathogenesis (Ma et al., 2019; Hong et al., 2024; Huang et al., 2023). These observations indicate that chronic BPA exposure could contributes to the development or exacerbation through immune and inflammatory dysregulation at the ocular surface. BPA is widely present in various media including indoor dust and settled particles (Zhu et al., 2023; Li et al., 2024; Pan et al., 2024; Lv et al., 2016). The ocular surface is directly open to the external environment. Therefore, corneal cells are continuously vulnerable to surrounding toxins. Meanwhile, increasing evidence demonstrates that common environmental pollutants can directly disrupt ocular surface homeostasis. For example, certain atmospheric contaminants alter the corneal epithelium and cause a significant loss of conjunctival cells (Torricelli et al., 2014; Yang et al., 2019). These parallel findings justify the evaluation of whether bisphenol A induces similar toxicity in corneal epithelial cells. This context provides a logical basis for utilizing a direct cellular model in our study. However, systematic investigation of the molecular interplay between BPA exposure and DED is currently lacking.

Given the complexity of both BPA toxicity and DED pathogenesis, conventional reductionist approaches are inadequate for capturing system-level interactions between environmental exposure and disease. Network toxicology integrates bioinformatics and multi-omics data to construct compound–target–pathway–disease networks and identify key molecular mediators (Sturla et al., 2014; Zhang et al., 2020). We designed a framework incorporating network toxicology, Mendelian randomization, and experimental validation (Karthikeyan et al., 2019; Li et al., 2025). This approach serves to propose potential hypotheses and provide preliminary validation rather than definitive proof of causality. This multi-level strategy aims to elucidate the molecular mechanisms between BPA exposure and DED and to provide mechanistic insight into the environmental contribution to ocular surface disease.

## 2 Materials and methods

### 2.1 Toxicity analysis

The Simplified Molecular Input Line Entry System (SMILES) notation of bisphenol A was obtained from PubChem and submitted to ProTox 3.0 to predict molecular initiating events, target pathway activities, and metabolic specificities. ADMETlab 3.0 was utilized to assess multidimensional safety endpoints.

### 2.2 Target prediction for BPA and DED

Experimentally validated chemical target interactions from the Comparative Toxicogenomics Database were prioritized as the primary candidates. Supplementary predictions were obtained from SwissTargetPrediction, PharmMapper, ChEMBL, STITCH, and SEA, with analyses restricted to *Homo sapiens*. Target identifiers were standardized using UniProt, and duplicate entries were removed after integration. These candidates were subsequently filtered using corneal tissue specific differentially expressed genes to identify targets with disease relevance. DED associated genes were collected from OMIM and GeneCards to generate the unified disease target list.

### 2.3 Identification of DED-associated differentially expressed genes

DED-related transcriptomic data were acquired from the Gene Expression Omnibus (GEO) platform. Two datasets were incorporated: GSE252984 (platform GPL24247), containing 4 control corneal samples and 4 DED corneal samples, and GSE208297 (platform GPL24247), comprising 5 control corneal samples and 5 DED corneal samples. Raw expression profiles were analyzed in R with the limma package. Duplicate probes were removed, and expression values were normalized with the normalizeBetweenArrays function. For datasets generated on the same platform, ComBat from the sva package was used to correct batch effects. To validate this correction, principal component analysis was performed (Supplementary Figure S1). Differentially expressed genes (DEGs) analysis was performed with limma, and genes meeting the criteria of adjusted *P* < 0.05 and |log_2_ fold change| > 1 were retained. Mouse genes were converted to their corresponding human orthologs using the NCBI HomoloGene database.

### 2.4 Functional and pathway enrichment analysis

Gene Ontology (GO) and Kyoto Encyclopedia of Genes and Genomes (KEGG) enrichment analyses were applied to examine functional characteristics of the identified target genes (*P* value <0.05). GO analysis was used to assess enrichment in cellular components, molecular functions, and biological processes. KEGG analysis was subsequently conducted to identify signaling pathways potentially related to BPA involvement in DED.

### 2.5 Protein–protein interaction (PPI) network construction and core target screening

PPI analysis was carried out with the STRING database (Szklarczyk et al., 2023; Chi et al., 2024). Target genes were uploaded under a *Homo sapiens* constraint, with an interaction confidence threshold set at 0.4. PPI network visualization and analysis were carried out in Cytoscape (Majeed and Mukhtar, 2023; Chen T. et al., 2025). We employed the CytoHubba tool to determine core genes in the network based on several topological metrics, and genes that ranked highly across multiple methods were selected.

### 2.6 Machine learning–based identification of core genes

An integrated strategy involving Random Forest, Support Vector Machine (SVM), and Least Absolute Shrinkage and Selection Operator (LASSO) regression was executed to screen for core genes associated with BPA-related DED. The random forest model was implemented using the “randomForest” package, and gene importance was evaluated using the Gini index. For SVM analysis, a classifier incorporating a radial basis function kernel was established via the “caret” environment, and feature selection was performed by SVM-RFE with 5-fold cross-validation. LASSO regression was conducted using the “glmnet” packagewith the optimal λ identified through 5-fold cross-validation. Genes with non-zero coefficients were selected. The intersection of three methods was selected for downstream analyses.

### 2.7 Mendelian randomization

Two-sample Mendelian randomization analysis was used to examine the potential causal effect of candidate gene expression on the phenotype. Genetic variants associated with gene expression were obtained from expression quantitative trait loci (eQTL) data sourced from whole blood samples in the eQTLGen consortium under the dataset identifier eqtl a ENSG00000137752. Outcome-related effects were derived from genome-wide association study (GWAS) summary statistics (GCST90043801). All datasets were publicly available. Single nucleotide polymorphisms (SNPs) significantly associated with the exposure were used as instrumental variables (*P* < 5 × 10^−8^). Independence among instruments was ensured by linkage disequilibrium (LD) clumping with an r^2^ threshold of 0.001 and a 10 kb window. Instrument strength was evaluated based on the *F*-statistic, with *F*-statistic >10 considered indicative of sufficient strength. The Inverse Variance Weighted (IVW) served as the primary method for causal estimation. Pleiotropy, heterogeneity, and the influence of individual SNPs were assessed using MR-Egger regression, Cochran’s Q statistics, and leave-one-out analyses. Analysis was conducted with the TwoSampleMR package (v0.5.7) in R.

### 2.8 Gene set enrichment analysis

GSEA was applied to further characterize biological processes and pathways associated with hub genes. Analysis was carried out with the “clusterProfiler” package and the “m5.go.v2025.1.Mm.symbols.gmt” gene set from Molecular Signatures Database (MSigDB). Genes were ranked by Spearman correlation coefficients, and significant enrichment was defined by false discovery rate (FDR) < 0.25 and |normalized enrichment score (NES)| > 1 (*P* < 0.05).

### 2.9 Immune infiltration analysis

Gene expression profiles from DED and control samples were analyzed to estimate the composition of immune cell with CIBERSORT. Statistical analysis was performed to identify cell populations showing proportional variations between the studied conditions (*P* < 0.05). Associations between immune cell proportions and core biomarkers were analyzed using correlation analysis.

### 2.10 Molecular docking analysis and molecular dynamics simulation

We conducted computational docking to evaluate the spatial engagement between BPA and the key target. An analytical protocol was adopted where the ligand retained its conformational freedom against a static macromolecular receptor. The spatial conformation of the target was sourced from the AlphaFold repository. Protein structure was prepared using PyMOL (version 3.1) by removing co-crystallized ligands and water molecules. The resulting structure was optimized using AutoDock Tools and AutoDock Vina (version 1.5.6). For each protein, a docking grid covering the predicted binding site was defined, and ten independent docking runs were conducted. Docking results were evaluated based on binding conformations and binding energy. Interactions were visualized using PyMOL. To validate the thermodynamic stability of the docked complex under physiological conditions, a one hundred nanosecond molecular dynamics simulation was executed using the Gromacs 2025 software package. The protein was parameterized using the AMBER14SB force field, and the ligand topology was constructed using the GAFF2 force field. The topology files were merged to ensure zero atom type conflicts. Periodic boundary conditions were implemented, placing the complex in a cubic box with a minimum distance of 1.2 nm from the solute to the box wall. The system was solvated using the TIP3P water model. Sodium and chloride ions were added at a concentration of 0.15 M to neutralize the net charge and mimic the physiological environment. Following energy minimization, the system underwent NVT and NPT equilibrations with a coupling constant of 0.1 picoseconds for a total duration of 2 nanoseconds. The production molecular dynamics run was carried out at a constant temperature of 310 K and a constant pressure of 1 bar for one hundred nanoseconds, employing a time step of two femtoseconds. Trajectory properties including root mean square deviation, root mean square fluctuation, radius of gyration, solvent accessible surface area, and hydrogen bond count were calculated using standard Gromacs utilities. The average binding free energy and individual residue energy decompositions were calculated using the MMPBSA method over the stable equilibrium trajectory. The Gibbs free energy landscape was plotted based on the first two principal components to characterize the thermodynamic equilibrium states.

### 2.11 Cell culture and treatment

Human immortalized corneal epithelial cells (HCECs; Procell, Wuhan, China) were cultured in DMEM/F12 medium (Meilunbio, Dalian, China) supplemented with 10% fetal bovine serum (FBS) and 1% penicillin-streptomycin. Cultures were maintained in conventional cell culture incubators (37 °C, 5% CO_2_). Cells reached 80%–85% confluence before the medium was replaced with concentrations of bisphenol A (BPA; TargetMol, Shanghai, China) at 0.1, 1, 10, or 100 μM. Cells were harvested after 24 h of exposure. To evaluate the role of caspase-1, the inhibitor Ac-YVAD-CHO (10 μM; TargetMol) was added to the medium during BPA treatment. Control groups received an equivalent volume of DMSO as a vehicle.

### 2.12 Cell Counting Kit-8 (CCK-8) assay

Cellular survival was quantified via the CCK-8 assay. HCECs were seeded into 96-well plates and incubated for 24 h for stable adherence. Following exposure to various concentrations of BPA for another 24 h, the culture medium was replaced with 100 μL of fresh medium containing 10 μL of CCK-8 solution (Solarbio, Beijing, China). After a 2 h incubation in the dark, the optical density (OD) at 450 nm was measured.

### 2.13 Lactate dehydrogenase (LDH) release assay and propidium iodide (PI) staining assay

Cytotoxicity was quantified by measuring the release of LDH into the culture supernatant using an LDH assay kit (Meilunbio, Dalian, China). High-control wells were treated with 10 μL of Lysis Buffer 30 min prior to the end of the 24 h BPA treatment. After centrifugation at 250 × g for 2 min, 100 μL of the supernatant was transferred to a new plate and mixed with 100 μL of LDH reaction mixture. Following a 30 min incubation at room temperature, the reaction was stopped, and absorbance was measured at 490 nm.

For the assessment of cell membrane permeability, a propidium iodide (PI) reagent (Meilunbio, Dalian, China) was employed at a working concentration of 20 μg/mL. The experimental treatment of human corneal epithelial cells was identical to the conditions described for the LDH release assay. Following the exposure window, the cells were incubated with the propidium iodide working solution at 37 °C for 20 min in the dark. Subsequently, nuclei were counterstained with DAPI (Solarbio) for 5 min. High resolution fluorescence images were captured utilizing an inverted fluorescence microscope, and the proportion of propidium iodide positive cells was quantified using ImageJ software.

### 2.14 Reactive oxygen species (ROS) detection

Intracellular ROS levels were evaluated using the fluorescent probe DCFH-DA. HCECs were seeded in 12-well plates and subjected to the indicated treatments (100 μM BPA with or without 10 μM Ac-YVAD-CHO) for 24 h. Cells were washed with PBS and incubated with 10 μM DCFH-DA for 20 min at 37 °C in the dark. After three washes with serum-free medium to remove the extracellular probe, cells were fixed with 4% paraformaldehyde for 15 min. Nuclei were counterstained with DAPI (Solarbio) for 5 min at room temperature. Fluorescence images were captured using an inverted fluorescence microscope. Fluorescence intensity was quantified using ImageJ software. A uniform threshold setting was applied consistently across all captured images.

### 2.15 RNA isolation and quantitative real-time PCR (qRT-PCR)

Total RNA was extracted from HCECs using a column-based RNA isolation kit (Vazyme, Nanjing, China). One microgram of total RNA was reverse-transcribed into cDNA using the HiScript III RT SuperMix (Vazyme). Quantitative real-time PCR was performed on a Bio-Rad CFX96 real-time PCR system (Bio-Rad, Hercules, CA, USA) using the ChamQ Universal SYBR qPCR Master Mix (Vazyme). The thermal cycling protocol consisted of an initial denaturation at 95 °C for 30 s, followed by 40 cycles of denaturation at 95 °C for 10 s, and annealing or extension at 60 °C for 30 s. The relative mRNA expression levels of *caspase-1*, *GSDMD*, *IL-1β,* and *IL-18* were calculated using the 2^−ΔΔCt^ method. β-actin was used as the endogenous control for normalization. The primer sequences used are as follows (Table 1).

**TABLE 1:** Information of primers used for RT-qPCR.

Target gene	Forward	Reverse
GSDMD	5′-ACT​GAG​GTC​CAC​AGC​CAA​GAG​G-3′	5′-GCC​ACT​CGG​AAT​GCC​AGG​ATG-3′
caspase-1	5′-ATA​CAA​CCA​CTC​GTA​CAC​GTC​TTG​C-3′	5′-TCC​TCC​AGC​AGC​AAC​TTC​ATT​TCT-3′
IL-1β	5′-TCG​CAG​CAG​CAC​ATC​AAC​AAG-3′	5′-TCC​ACG​GGA​AAG​ACA​CAG​GTA​G-3′
IL-18	5′-GTC​GCA​GAT​GGC​TCT​TTG​CT-3′	5′-TGC​CAA​AGT​AAT​CTG​ATT​CCA​GGT-3′
β-actin	5′-TGG​CAC​CCA​GCA​CAA​TGA​A-3′	5′-CTA​AGT​CAT​AGT​CCG​CCT​AGA​AGC​A-3′

All primer sequences listed above correspond to *Homo sapiens* transcripts.

### 2.16 Western blot analysis

Human immortalized corneal epithelial cells were lysed with RIPA lysis buffer (Solarbio, Beijing, China). Protein concentrations were measured by BCA assay, mixed with SDS sample buffer, and boiled for 10 min at 95 °C. The protein samples were separated by 10%–12% sodium dodecyl sulfate polyacrylamide gel electrophoresis (SDS PAGE) and transferred onto a polyvinylidene difluoride (PVDF) membrane. The membranes were blocked with 5% skimmed milk at room temperature for 2 h and then incubated with primary antibodies diluted by antibody dilution buffer (Beyotime, Shanghai, China) at 4 °C overnight. Primary antibodies obtained from Wanlei Bio (Shenyang, China) included those against cleaved caspase 1, GSDMD, mature IL 1β, mature IL 18, and β-actin. The following day, the membranes were washed with 1× Tris buffered saline and Tween 20 (TBST) three times and incubated with secondary antibodies at room temperature for 1 h. Protein expression levels were tested with a chemiluminescence assay (Thermo Fisher Scientific, Waltham, MA, United States). The ratio of the gray value of the target band to β-actin was representative of the relative protein expression.

### 2.17 Statistical analyses

Statistical analyses and data visualization were performed using R software (version 4.4.2) and GraphPad Prism 10. All quantitative experiments were performed independently at least three times. Data are presented as the mean ± standard deviation (SD). Differences between two groups were evaluated using Student’s t-test, while comparisons among multiple groups were assessed via one-way analysis of variance (ANOVA). Statistical significance was defined as *P* < 0.05.

## 3 Result

### 3.1 Toxicity prediction

**FIGURE 1:**
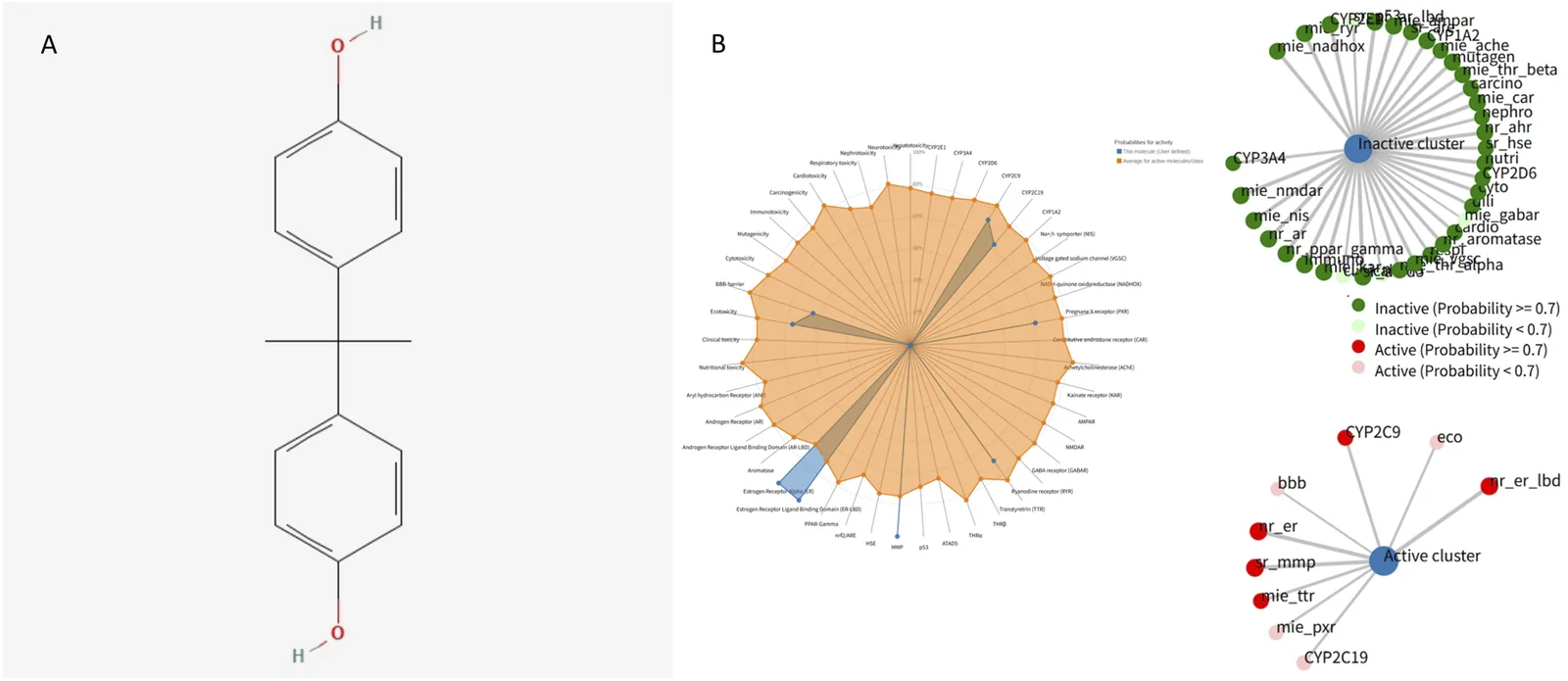
Molecular Structure and Toxicity Map of Bisphenol A. **(A)** Molecular formula of BPA; **(B)** Toxicity map of BPA.

**FIGURE 2:**
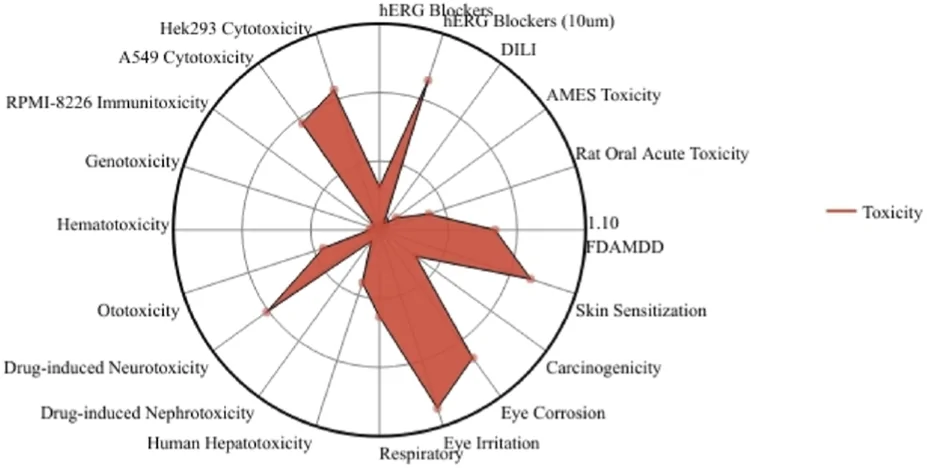
Toxicity radar chart.

BPA exhibited blood-brain barrier (BBB) permeability and ecotoxicity in ProTox 3.0 simulations. Molecular initiating events suggested high binding probabilities for transthyretin (TTR) and pregnane X receptor (PXR). Pathway analysis indicated targeted activity toward estrogen receptor alpha (ERα) and interference with mitochondrial membrane potential (MMP). BPA was further categorized as a potential substrate for the cytochrome P450 enzymes CYP2C19 and CYP2C9 (Table 2; Figure 1). Multidimensional toxicity assessment via ADMETlab 3.0 revealed high risks for ocular irritation, corrosion, and skin sensitization. Predicted systemic toxicities included hERG channel blockade, significant neurotoxicity, and potent cytotoxicity against A549 and HEK293 cell lines (Table 3; Figure 2). These findings define BPA as a hazardous agent with specific potential for ocular surface damage.

**TABLE 2:** Toxicity prediction-ProTox 3.0

Classification	Target	Prediction	Probability
Toxicity end points	Ecotoxicity	Active	0.63
Toxicity end points	BBB-barrier	Active	0.53
Molecular Initiating Events	Pregnane X receptor	Active	0.66
Molecular Initiating Events	Transtyretrin	Active	0.74
Tox21-Nuclear receptor signalling pathways	Estrogen Receptor Alpha	Active	1
Tox21-Nuclear receptor signalling pathways	Estrogen Receptor Ligand Binding Domain	Active	1
Tox21-Stress response pathways	Mitochondrial Membrane Potential	Active	1
Metabolism	CYP2C19	Active	0.68
Metabolism	CYP2C9	Active	0.77

**TABLE 3:** Toxicity prediction-Admetlab 3.0

Classification	Probability
hERG Blockers	0.228
hERG Blockers (10um)	0.837
DILI	0.04
FDAMDD	0.614
Rat Oral Acute Toxicity	0.275
AMES Toxicity	0.109
Genotoxicity	0.043
Hematotoxicity	0.052
Ototoxicity	0.316
Drug-induced Neurotoxicity	0.742
Drug-induced Nephrotoxicity	0.074
Human Hepatotoxicity	0.295
Respiratory	0.462
Eye Irritation	0.998
Eye Corrosion	0.843
Carcinogenicity	0.239
Skin Sensitization	0.846
RPMI-8226 Immunitoxicity	0.032
A549 Cytotoxicity	0.701
Hek293 Cytotoxicity	0.785

### 3.2 Candidate target identification of DED induced by BPA

**FIGURE 3:**
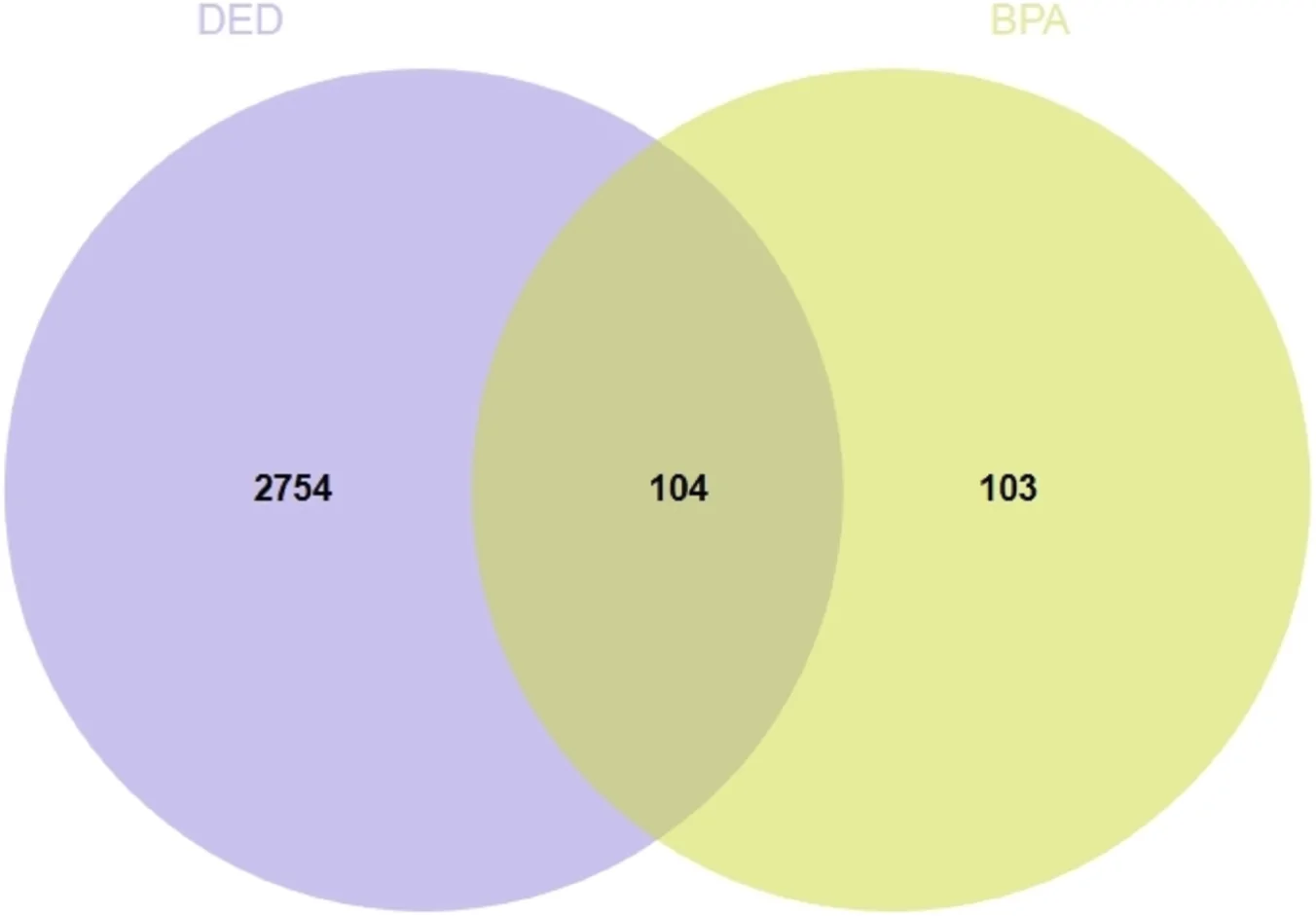
Venn diagram showing the overlap between targets of DED and BPA.

Prioritizing experimentally validated interactions in the Comparative Toxicogenomics Database and integrating complementary predictions from SwissTargetPrediction, PharmMapper, ChEMBL, STITCH, and SEA identified 207 unique BPA related targets. In parallel, 2,858 DED-associated genes were retrieved from OMIM and GeneCards. Overlap analysis between these two sets yielded 104 candidate targets (Figure 3).

**FIGURE 4:**
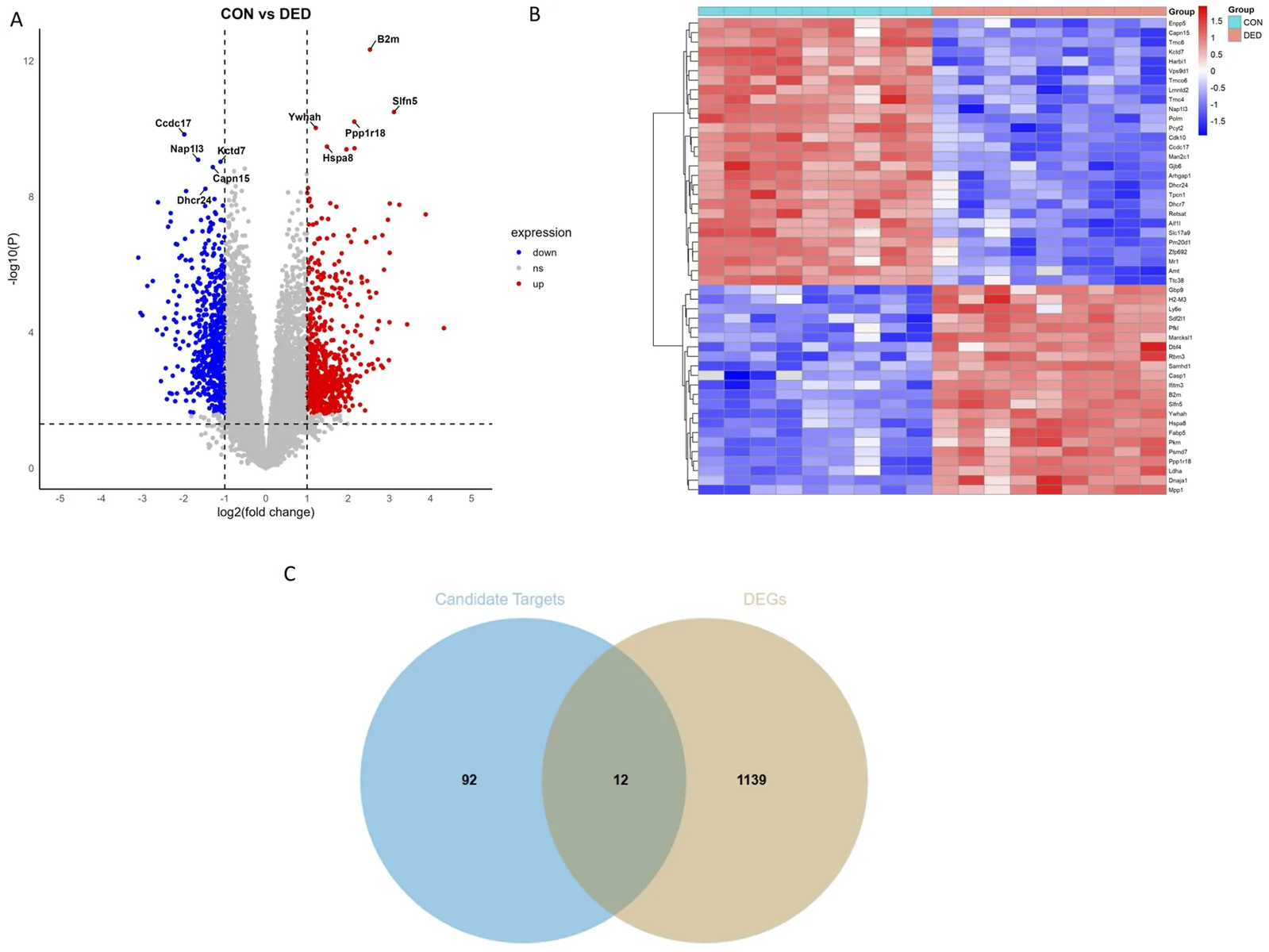
Identification of DEGs in dry eye disease. **(A)** Volcano plot; **(B)** Heatmap; **(C)** Venn diagram.

To refine these candidates based on disease-specific expression profiles, we analyzed the GSE252984 and GSE208297 datasets, identifying 1,282 DEGs (677 upregulated and 605 downregulated). These DEGs were visualized using a volcano plot (Figure 4A). A heatmap of the top 50 DEGs with the largest expression changes demonstrated distinct transcriptional differences between DED and control samples (Figure 4B). Mapping these sequences to human orthologs resulted in 1,151 genes. The final intersection of these 1,151 orthologs with the 104 initial candidates prioritized 12 core targets (*CA2*, *GSR*, *PTGS2*, *ADORA1*, *PTPRC*, *CASP1*, *TNF*, *BCL2*, *CASP3*, *VEGFA*, *CD36*, and *MYB*) for further investigation (Figure 4C).

### 3.3 Functional enrichment analysis

**FIGURE 5:**
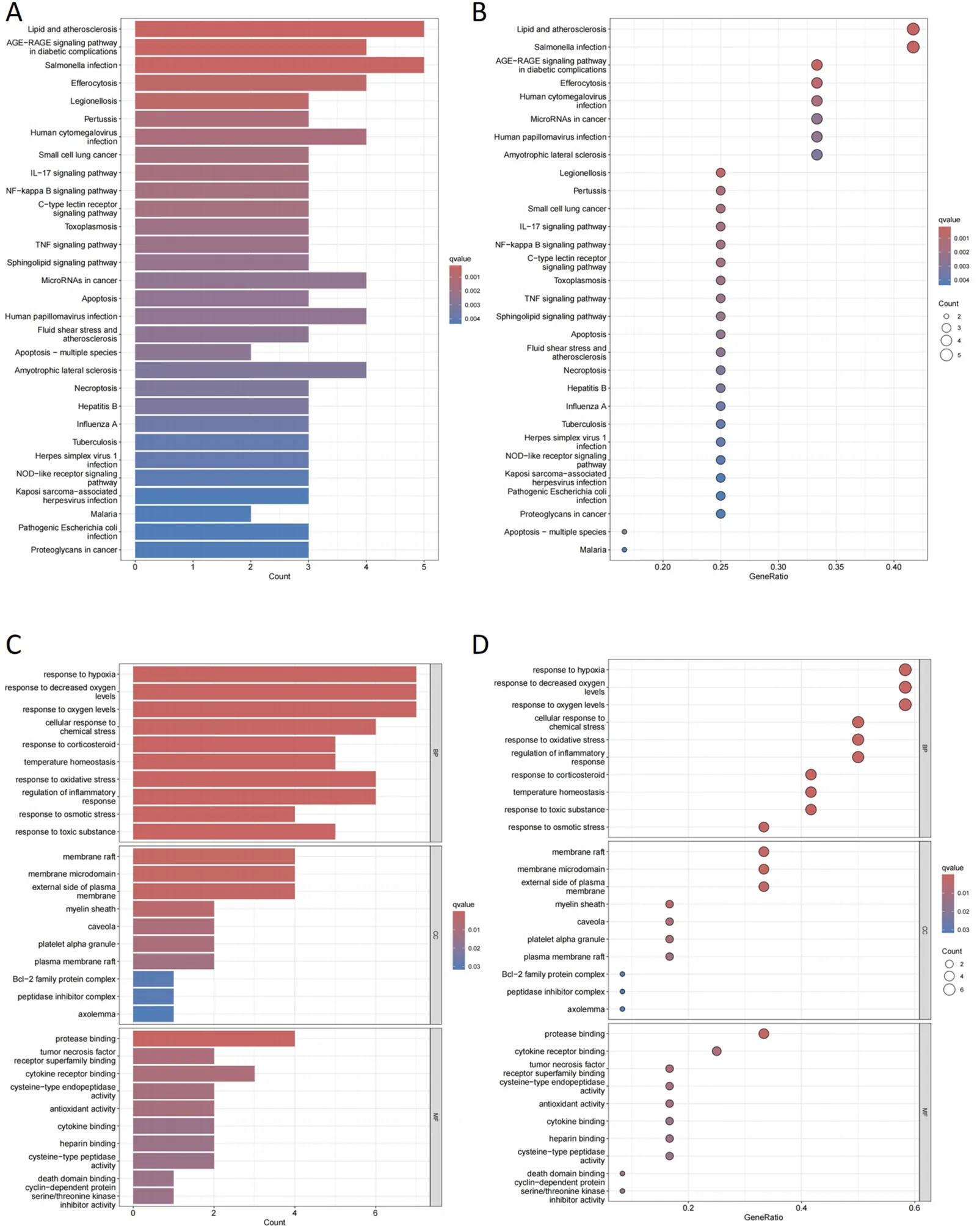
GO and KEGG Analysis. **(A)** and **(C)** Bar chart; **(B)** and **(D)** Bubble chart.

We subjected the overlapping genes to functional profiling within the R environment, referencing both the GO and KEGG databases. KEGG pathway analysis identified 72 significantly enriched pathways. The top 30 pathways (Figures 5A,B) were primarily associated with classical pro-inflammatory cascades (IL-17, TNF, and NF-κB signaling) and programmed cell death. Enrichment was also observed in stress and metabolic networks, such as the AGE-RAGE signaling pathway. GO enrichment analysis identified 1,421 significant terms, including 76 molecular functions (MFs), 24 cellular components (CCs), and 1,321 biological processes (BPs). As visualized in Figures 5C,D, BPs centered on responses to hypoxia, oxidative stress, and toxic substances. Cellular components mapped to the Bcl-2 family protein complex and membrane rafts, while molecular functions were characterized by cytokine receptor binding and antioxidant activity. These profiles indicate that the core targets mediate oxidative imbalance and aberrant inflammatory signaling at the ocular surface.

### 3.4 PPI network and identification of core targets

**FIGURE 6:**
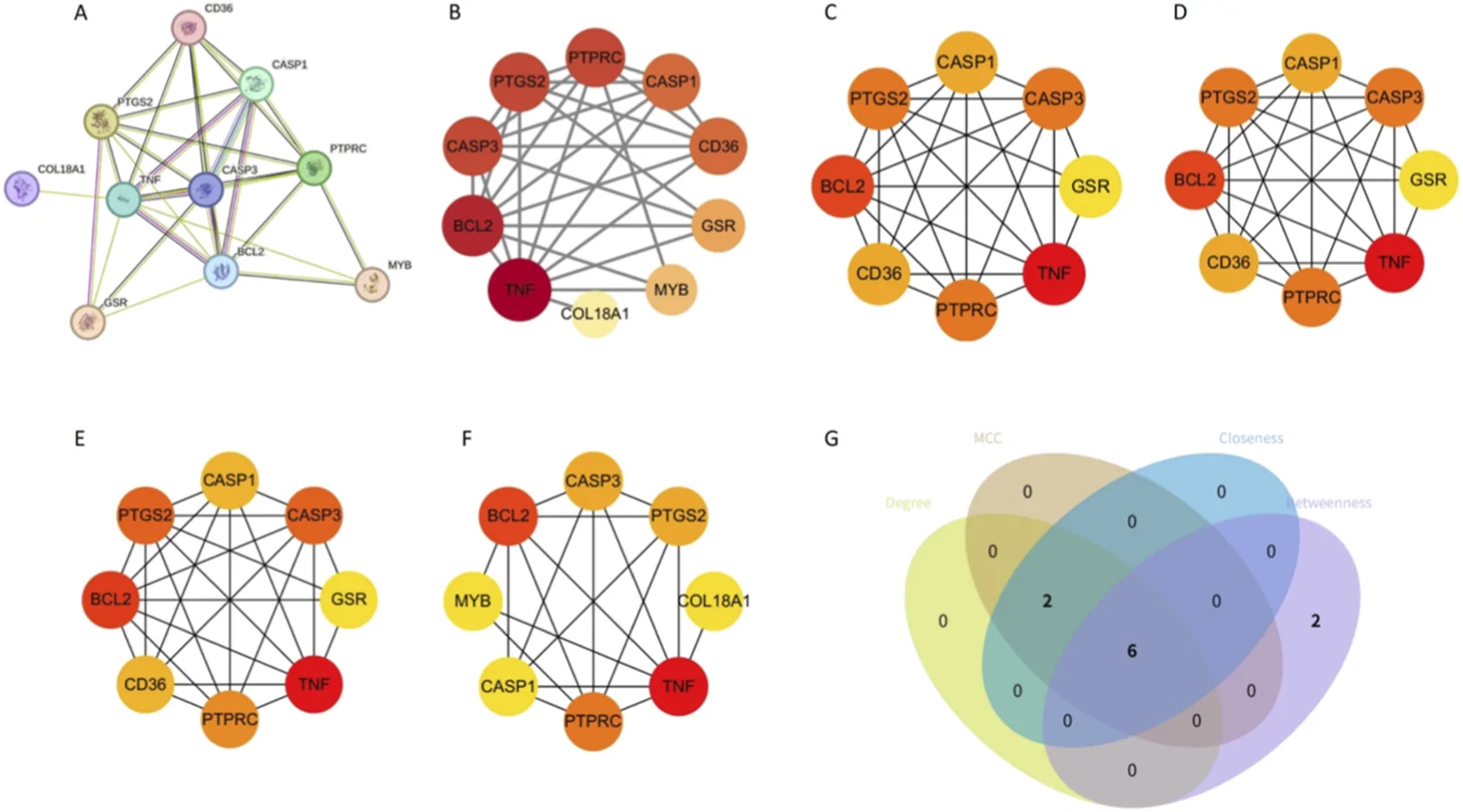
PPI network and topological screening of hub genes. **(A)** The PPI network for BPA targtes in DED. **(B)** PPI network in Cytoscape v3.10.0, showing proteins as nodes and interactions as edges. **(C–F)** Identification of the top eight targets based on four topological algorithms: degree **(C)**, MCC **(D)**, closeness **(E)**, and betweenness **(F)**. **(G)** Venn diagram.

To map the interaction landscape of the 12 candidate targets, we acquired the protein-protein crosstalk data from the STRING repository. PPI network was rendered in Cytoscape (Figures 6A,B). Network topology was evaluated utilizing four distinct algorithms: Degree, Maximum Neighborhood Component (MCC), Closeness, and Betweenness centrality (Figures 6C–F). The top eight nodes from each algorithmic ranking were intersected for further analysis (Figure 6G).

### 3.5 Machine learning–based identification of core genes

**FIGURE 7:**
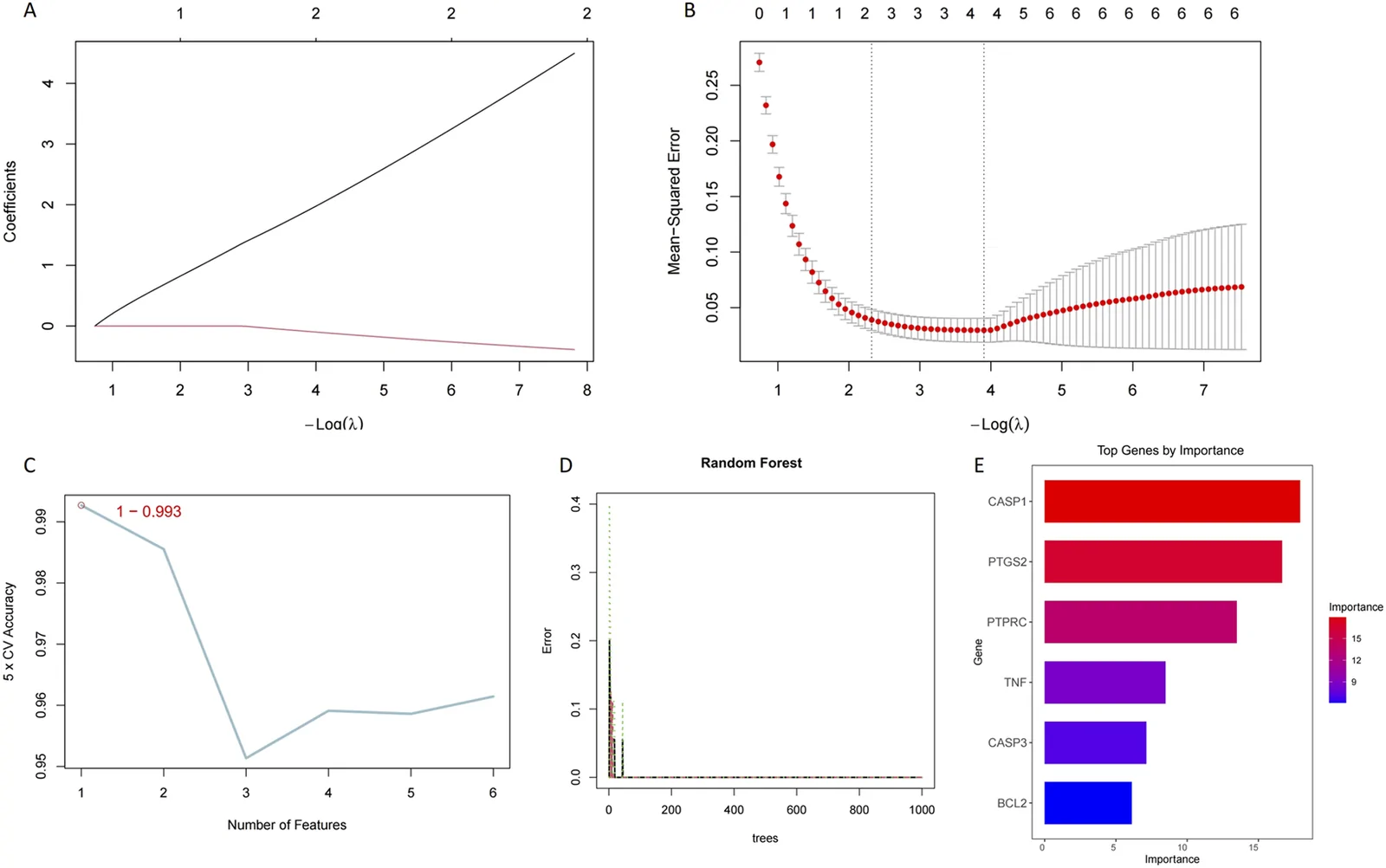
Core gene identification via machine learning algorithms for BPA-induced DED **(A,B)** LASSO algorithm. **(C)** SVM-RFE algorithm. **(D,E)** Random forest algorithm.

Three machine learning methods were applied. For the LASSO framework, 5-fold cross-validation was executed to determine the optimal regularization parameter (λ = 0.02019473) (Figures 7A,B), under which *CASP1* and *TNF* were retained. SVM-RFE analysis was subsequently performed to evaluate feature importance. The highest classification accuracy was achieved when one gene, *CASP1*, was retained (Figure 7C). Random forest analysis ranked genes according to their importance scores, with *CASP1* showing the highest score, followed by *PTGS2*, *PTPRC*, *TNF*, *CASP3*, and *BCL2* (Figures 7D,E). Results identified *CASP1* as the most prominent core gene associated with BPA-related DED.

### 3.6 Mendelian randomization

**FIGURE 8:**
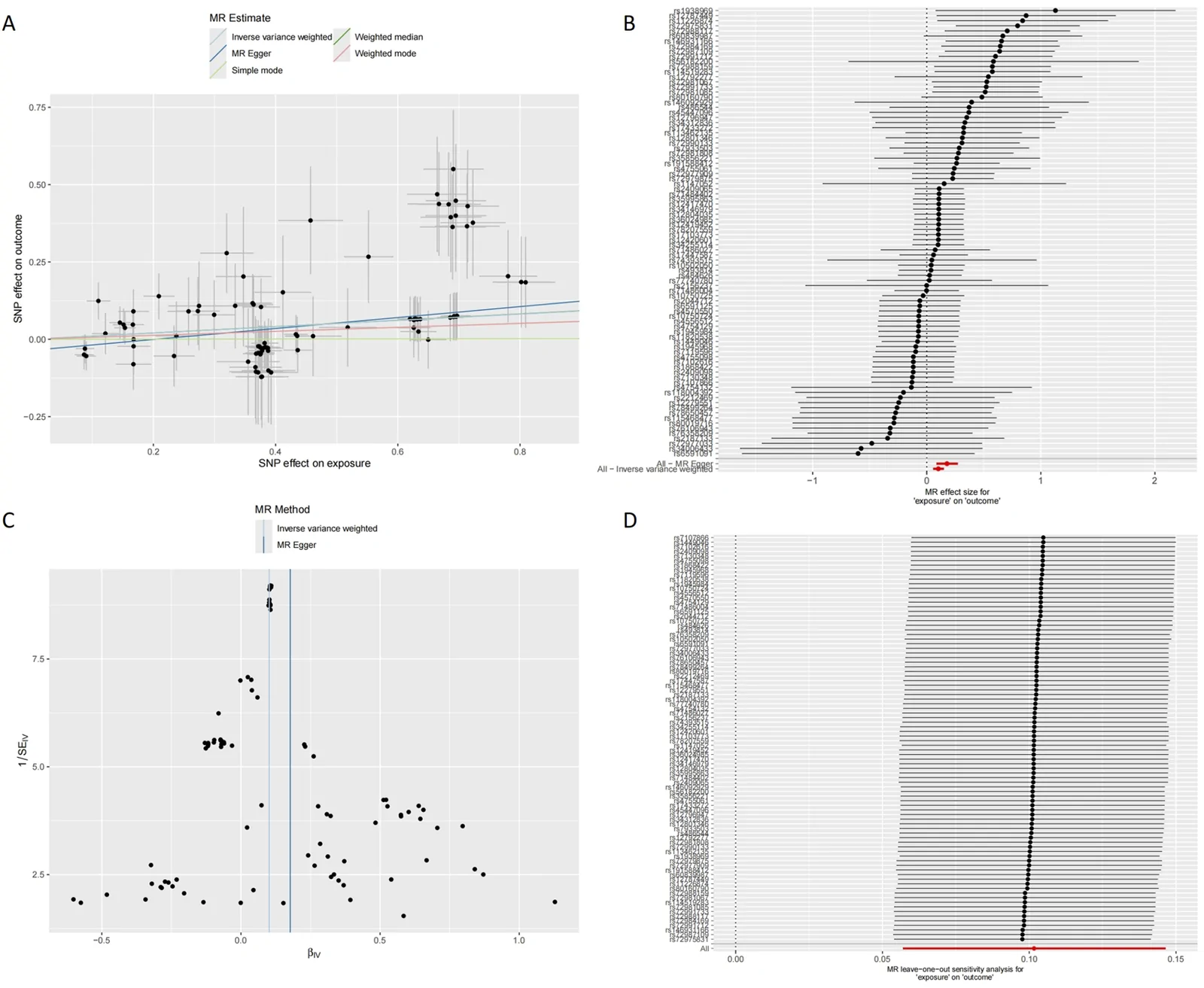
Mendelian randomization analyses assessing the causal effect of *CASP1* expression on dry eye disease. **(A)** Scatter plot illustrating the associations between single nucleotide polymorphism (SNP) effects on *CASP1* expression (exposure) and dry eye disease risk (outcome). Each point represents an individual SNP, with regression lines corresponding to different MR methods. **(B)** Forest plot showing the individual causal estimates for each SNP on dry eye disease, along with the pooled estimates derived from the IVW and MR-Egger methods. Horizontal lines indicate 95% confidence intervals. **(C)** Funnel plot assessing potential directional pleiotropy. **(D)** Leave-one-out sensitivity analysis.

MR analysis was used to evaluate the causal relationship between *CASP1* expression and DED risk, with *CASP1* expression as the exposure and DED as the outcome. Using the IVW method, higher *CASP1* expression was linked to a higher risk of DED (β = 0.102, SE = 0.023, *P* = 8.17 × 10^−6^), corresponding to an OR of 1.11 (95% CI: 1.06–1.16). Consistent effect estimates were observed using complementary MR methods. The weighted median method yielded an OR of 1.11 (95% CI: 1.04–1.17, *P* = 6.19 × 10^−4^), while MR-Egger regression showed an OR of 1.19 (95% CI: 1.09–1.31, *P* = 2.78 × 10^−4^) (Table 4; Figure 8). Sensitivity analyses showed no significant heterogeneity among instrumental variables (IVW Cochran’s Q = 103.66, *P* = 0.11; MR-Egger Q = 99.72, *P* = 0.15). The MR-Egger intercept test did not show presence of horizontal pleiotropy (intercept = −0.036, *P* = 0.069). Leave-one-out analysis showed that the association was not driven by individual SNPs.

**TABLE 4:** The Causal association between CASP1 expression and the risk of DED.

Method	nsnp	OR	P-value
MR Egger	88	1.193,815	2.78 × 10^−4^
Weighted median	88	1.107,859	4.12 × 10^−4^
Inverse variance weighted	88	1.107,001	8.17 × 10^−6^
Simple mode	88	1.002060	9.66 × 10^−1^
Weighted mode	88	1.066471	6.76 × 10^−2^

### 3.7 Gene set enrichment analysis

**FIGURE 9:**
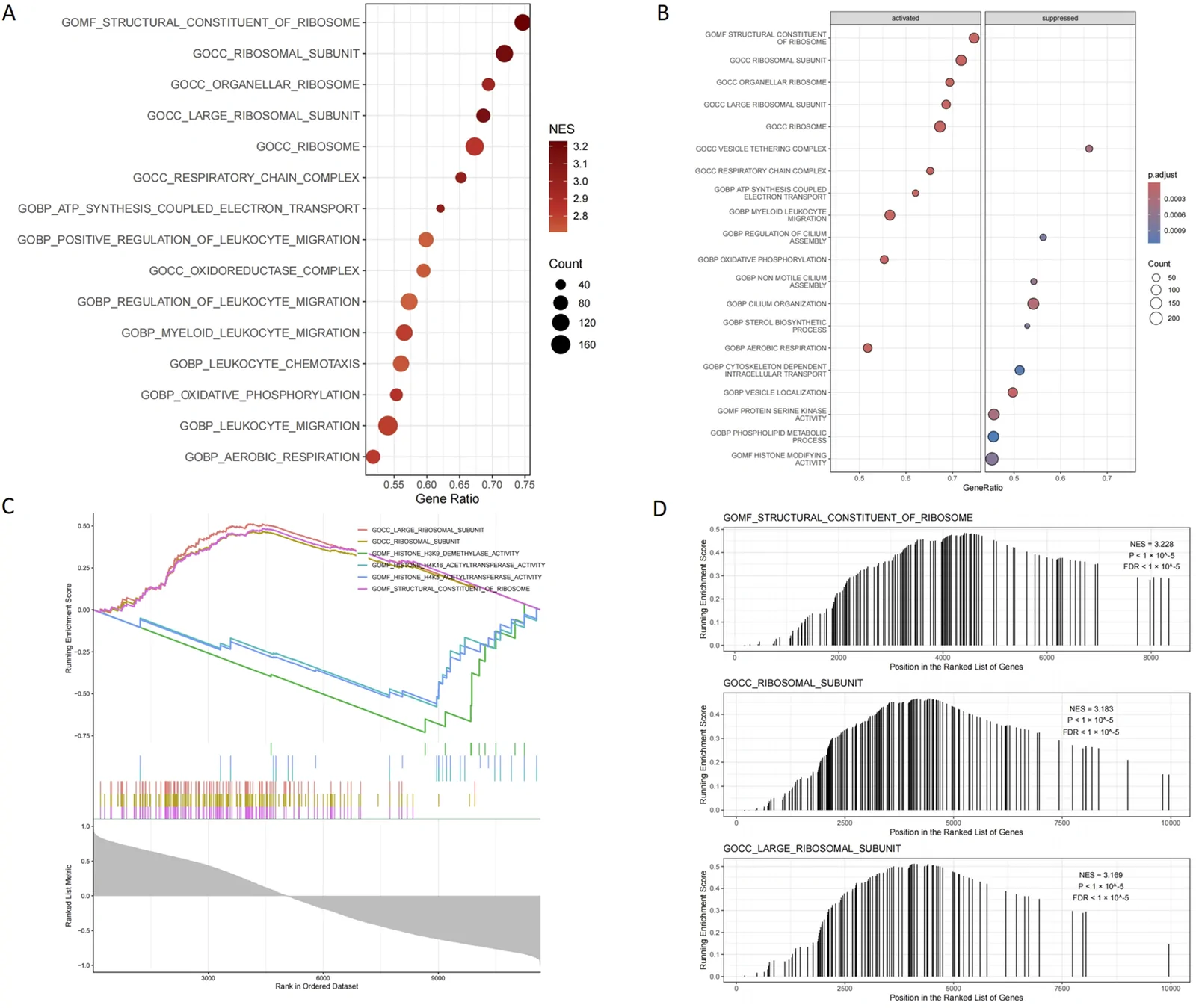
GSEA of *CASP1*-associated transcriptional programs. **(A)** Dot plot summarizing significantly enriched GO terms identified by GSEA based on genes ranked according to their Spearman correlation with *CASP1* expression. **(B)** Dot plot showing enriched GO terms stratified by the direction of enrichment. Pathways with positive and negative NES values are displayed separately **(C)** Combined visualization of positively and negatively enriched pathways. **(D)** Individual GSEA enrichment plots for representative top positively enriched pathways.

GSEA was performed based on gene rankings according to their Spearman correlation with *CASP1* expression across the transcriptome. This approach allowed the identification of coordinated pathway-level changes independent of arbitrary differential expression thresholds. The overall enrichment results are shown in Figure 9A. Genes positively correlated with *CASP1* expression were significantly enriched in pathways associated to mitochondrial energy metabolism and protein synthesis. These pathways included ATP synthesis coupled electron transport, aerobic respiration, respiratory chain complex organization and oxidative phosphorylation. Multiple ribosome-related gene sets, such as structural constituent of ribosome and ribosomal subunit, showed significant positive enrichment (Figure 9A). When enrichment results were examined by direction, metabolic and translational pathways were mainly positively enriched, whereas gene sets related to transcriptional regulation and RNA processing were negatively enriched (Figures 9B,C). Enrichment plots of representative gene sets further demonstrated strong enrichment of ribosome-associated pathways (Figure 9D). These findings indicate a distinct transcriptomic reprogramming coupled with *CASP1* activation, characterized by amplified energetic and translational activities alongside suppressed RNA metabolism.

### 3.8 Immune infiltration analysis

**FIGURE 10:**
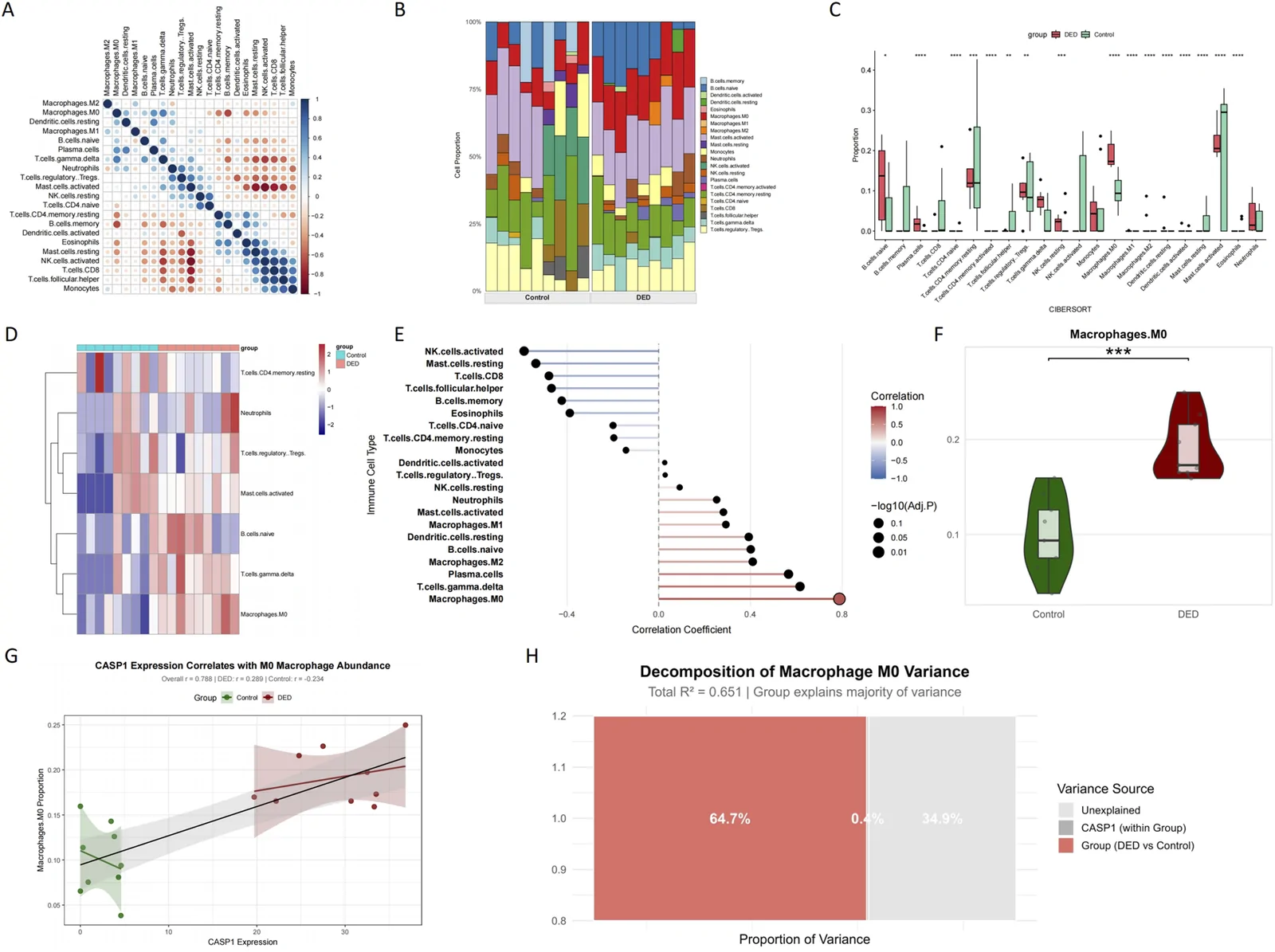
Immune infiltration analysis. **(A)** Pairwise correlation heatmap of immune cell proportions. **(B)** Immune cell composition in DED and control groups. **(C)** Differential abundance analysis of immune cell proportions. **(D)** Heatmap depicting the standardized abundance of immune cell types. **(E)** Bubble plot illustrating correlations between *CASP1* expression and immune cell proportions. Bubble size indicates adjusted significance level, and color represents correlation direction and magnitude. **(F)** Violin plot comparing Macrophages M0 proportions between control and DED groups. **(G)** The relationship between *CASP1* expression and Macrophages M0 proportion. **(H)** Variance decomposition of Macrophages M0 abundance based on a linear model.

Pairwise correlation analysis among immune cell subsets showed structured co-variation patterns across samples (Figure 10A). Immune cell composition differed between control groups and DED, revealing an overall redistribution of immune cell proportions (Figure 10B). Differential abundance analysis identified several immune cell types with significant differences between groups. Compared with control group, M0 macrophages were significantly increased in the DED group (*P* < 0.001; Figure 10C). Heatmap visualization further demonstrated consistently higher proportions of M0 macrophages in DED samples (Figure 10D). Across all samples, *CASP1* expression showed a positive correlation with M0 macrophage abundance (Figure 10E). M0 macrophage proportions were also significantly higher in DED samples than in controls on a per-sample basis (Figure 10F). However, this association dissipated upon group stratification, manifesting a classic Simpson’s paradox. Although a strong positive correlation was observed in the combined dataset (Pearson r = 0.788, *P* = 1.03 × 10^−4^), no significant correlation was detected within either the control group (r = −0.234, *P* = 0.544) or the DED group (r = 0.289, *P* = 0.450) (Figure 10G). Variance decomposition analysis indicated that disease status (DED vs. control) accounted for approximately 64.7% of the explained variance in M0 macrophage infiltration, whereas *CASP1* expression contributed minimally within groups (approximately 0.4%), with the remaining variance unexplained (Figure 10H).

### 3.9 Molecular docking analysis and molecular dynamics simulation

**FIGURE 11:**
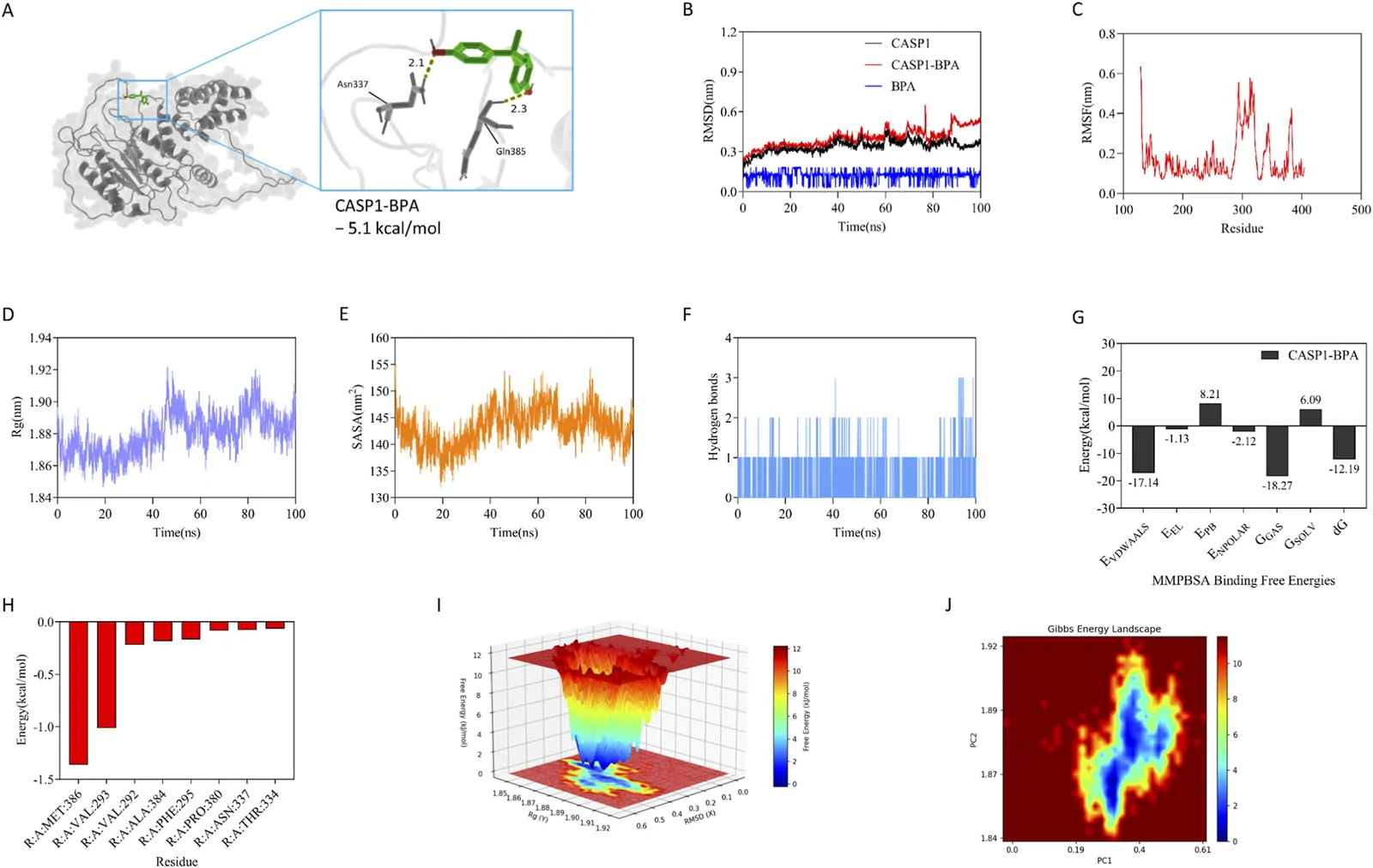
Molecular docking, molecular dynamics, and binding energy landscapes of the CASP1 BPA complex. **(A)** CASP1-BPA molecular docking diagram. **(B)** Root mean square deviation profiles of the protein, ligand, and complex over the one hundred nanosecond trajectory. **(C)** Root mean square fluctuation values per residue. **(D)** Radius of gyration showing structural compactness. **(E)** Solvent accessible surface area values. **(F)** Temporal distribution of intermolecular hydrogen bonds. **(G)** MMPBSA binding free energy components. **(H)** Decomposed binding energy contributions of key active site residues. **(I)** Three dimensional Gibbs free energy landscape showing the thermodynamic minimum valley. **(J)** Two dimensional Gibbs free energy landscape contour map.

Molecular docking predicted a potential initial non covalent engagement between BPA and the active site of CASP1, generating a docking score of minus 5.1 kcal per mol (Figure 11A). In this static snapshot, the hydroxyl groups of the ligand establish primary hydrogen bonds with the residues Asn337 and Gln385. To characterize the dynamic evolution and thermodynamic stability of this interaction under simulated physiological environments, a one hundred nanosecond molecular dynamics trajectory was analyzed. The root mean square deviation curves demonstrate that the complex achieves structural equilibrium rapidly after 20 ns. The backbone coordinates of the receptor and the ligand protein complex remained highly synchronized, and the ligand root mean square deviation fluctuated within an exceptionally low range, indicating that BPA remains securely anchored within the pocket without undergoing dissociation or substantial displacement (Figure 11B). Local residue flexibility analysis via root mean square fluctuations confirmed high rigidity across the core functional domains of the protein, while flexible behavior was confined to the terminal loops (Figure 11C). The radius of gyration and solvent accessible surface area values remained remarkably constant throughout the simulation window, confirming that the protein maintains a tight fold and preserves its hydrophobic core during ligand accommodation (Figures 11D,E). This structural integrity is sustained by a continuous network of intermolecular hydrogen bonds (Figure 11F). The total binding free energy calculated via the MMPBSA method was minus 12.19 kcal per mol, confirming a highly favorable and spontaneous thermodynamic process. Energy component analysis revealed that van der Waals interactions and gas phase energies serve as the principal driving forces for complex stabilization, whereas polar solvation energy acts as the primary destabilizing barrier (Figure 11G). Decomposition of the binding energy on a per residue basis indicated that residues MET386, VAL293, and VAL292 contributed the highest stabilizing energy (Figure 11H). Notably, while initial static docking highlighted hydrogen bonding at Asn337, the dynamic trajectory reveals an induced fit adaptation where the binding pocket reorganizes to optimize wider hydrophobic contacts anchored by MET386 and VAL293. Gibbs free energy landscape analysis further verified this behavior, mapping a single, continuous, deep blue energy minimum valley (Figures 11I,J). The lack of discrete high energy states demonstrates that the complex resides in a single dominant, thermodynamically stable conformation during the simulation.

### 3.10 *In vitro* validation of BPA-induced cytotoxicity and molecular mechanisms

#### 3.10.1 BPA reduces HCEC viability and induces cytotoxicity

**FIGURE 12:**
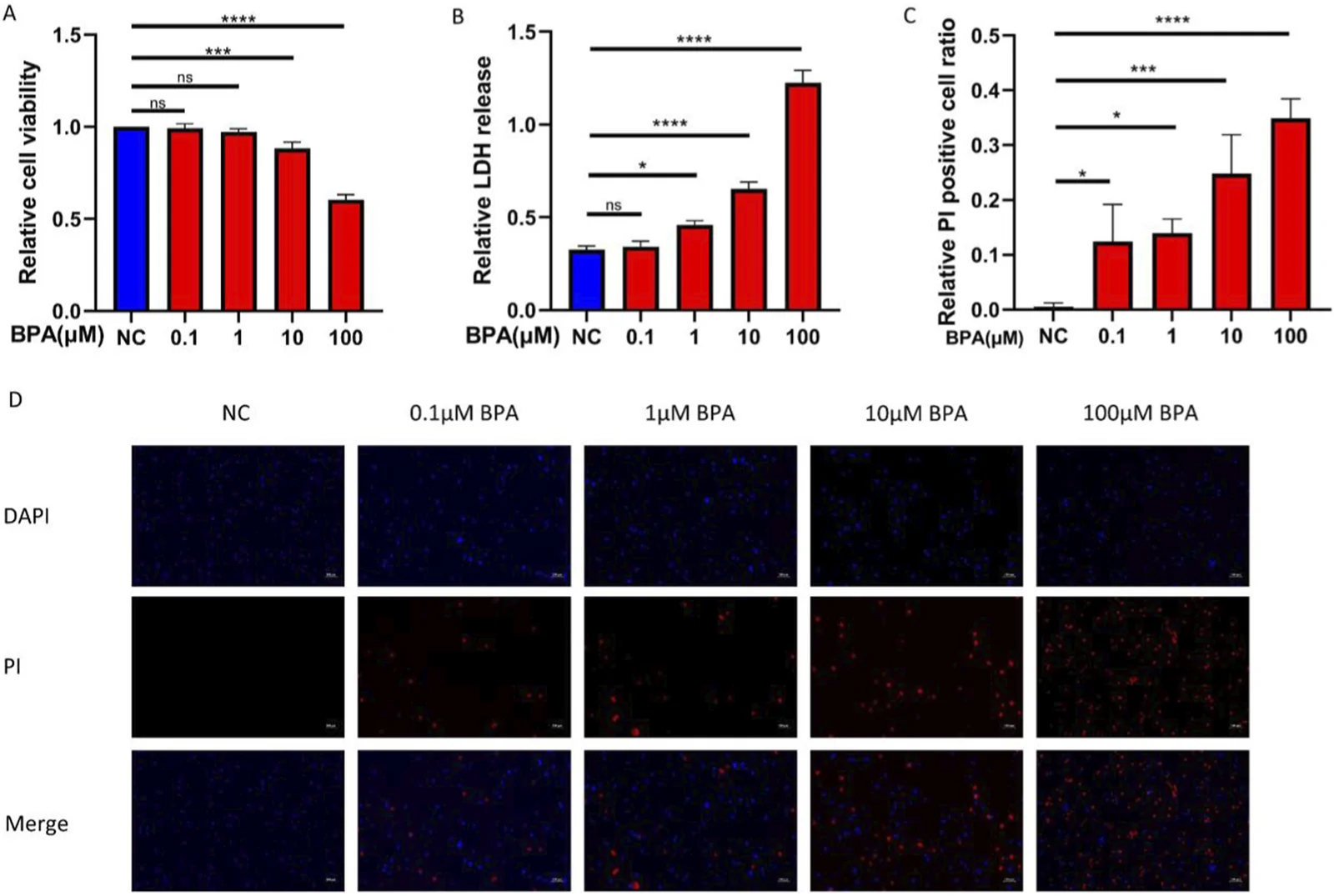
BPA-induced cytotoxicity in human corneal epithelial cells (HCECs). **(A)** Cell viability measured by CCK-8 assay following 24 h exposure to BPA at 0.1, 1, 10, and 100 μM. **(B)** Lactate dehydrogenase (LDH) release into the culture supernatant. **(C)** Statistical quantification of the relative propidium iodide (PI) positive cell ratio illustrating dose-dependent membrane compromise. **(D)** Representative fluorescence micrographs showing DAPI (blue), PI (red), and merged channels in HCECs across the indicated concentration gradients. Scale bar = 100 μm (as indicated in the panels). All data are expressed as mean ± SD (n = 3). *P < 0.05, **P < 0.01, ***P < 0.001, and ****P < 0.0001 vs. the control group.

CCK-8, LDH release and propidium iodide staining assays were performed to evaluate the cytotoxicity of BPA (0.1, 1, 10, and 100 μM) on HCECs following a 24 h exposure. Cell viability remained unchanged at 0.1 and 1 μM (*P* > 0.05). However, LDH release increased significantly starting at 1 μM (*P* < 0.05). At 10 and 100 μM, HCECs exhibited a sharp decline in viability alongside a peak increase in LDH leakage (*P* < 0.0001; Figures 12A,B). Control cultures displayed negligible red fluorescence, whereas exposure to BPA led to a dose dependent surge in the relative propidium iodide positive cell ratio, confirming widespread membrane compromise that closely mirrors the LDH leakage profiles (Figures 12C,D). The 100 μM concentration stably induced significant membrane damage and was selected for subsequent mechanism experiments.

#### 3.10.2 BPA triggers intracellular ROS accumulation

**FIGURE 13:**
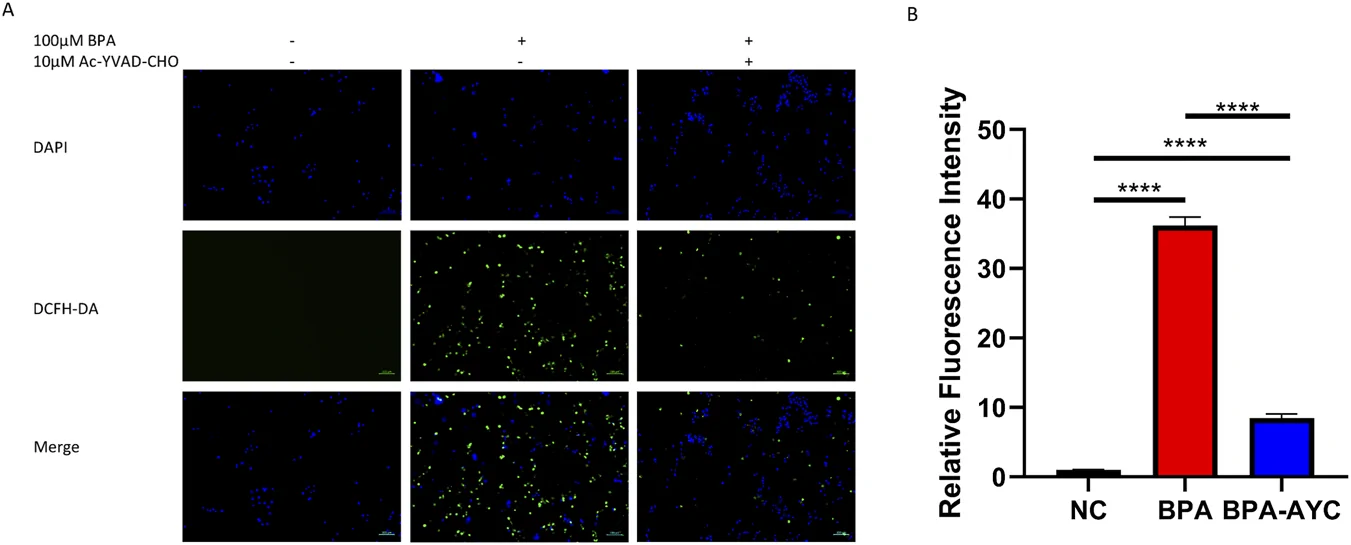
Promotion of intracellular ROS accumulation by BPA. **(A)** Fluorescence images showing DCFH-DA staining (green) in HCECs treated with 100 μM BPA and the CASP1 inhibitor Ac-YVAD-CHO (10 μM). Nuclei were counterstained with DAPI (blue). Scale bar = 100 μm. **(B)** Statistical quantification of relative ROS levels. Data are presented as mean ± SD (n = 3). BPA exposure triggered a ROS surge, which was partially mitigated by CASP1 inhibition. ****P < 0.0001. The custom label BPA-AYC denotes human corneal epithelial cells co treated with 100 μM BPA and 10 μM Ac-YVAD-CHO.

Intracellular ROS levels were evaluated using the DCFH-DA probe. Control HCECs exhibited minimal background fluorescence. Exposure to 100 μM BPA for 24 h substantially intensified intracellular green fluorescence, and subsequent quantitative analysis showed a surge in relative ROS levels compared to the control group (*P* < 0.0001, Figure 13). Co-treatment with the CASP1 inhibitor Ac-YVAD-CHO attenuated this ROS accumulation. This indicates that BPA-induced oxidative imbalance in HCECs partially depends on anomalous CASP1 pathway activation.

#### 3.10.3 BPA activates CASP1-dependent pyroptosis and inflammation

**FIGURE 14:**
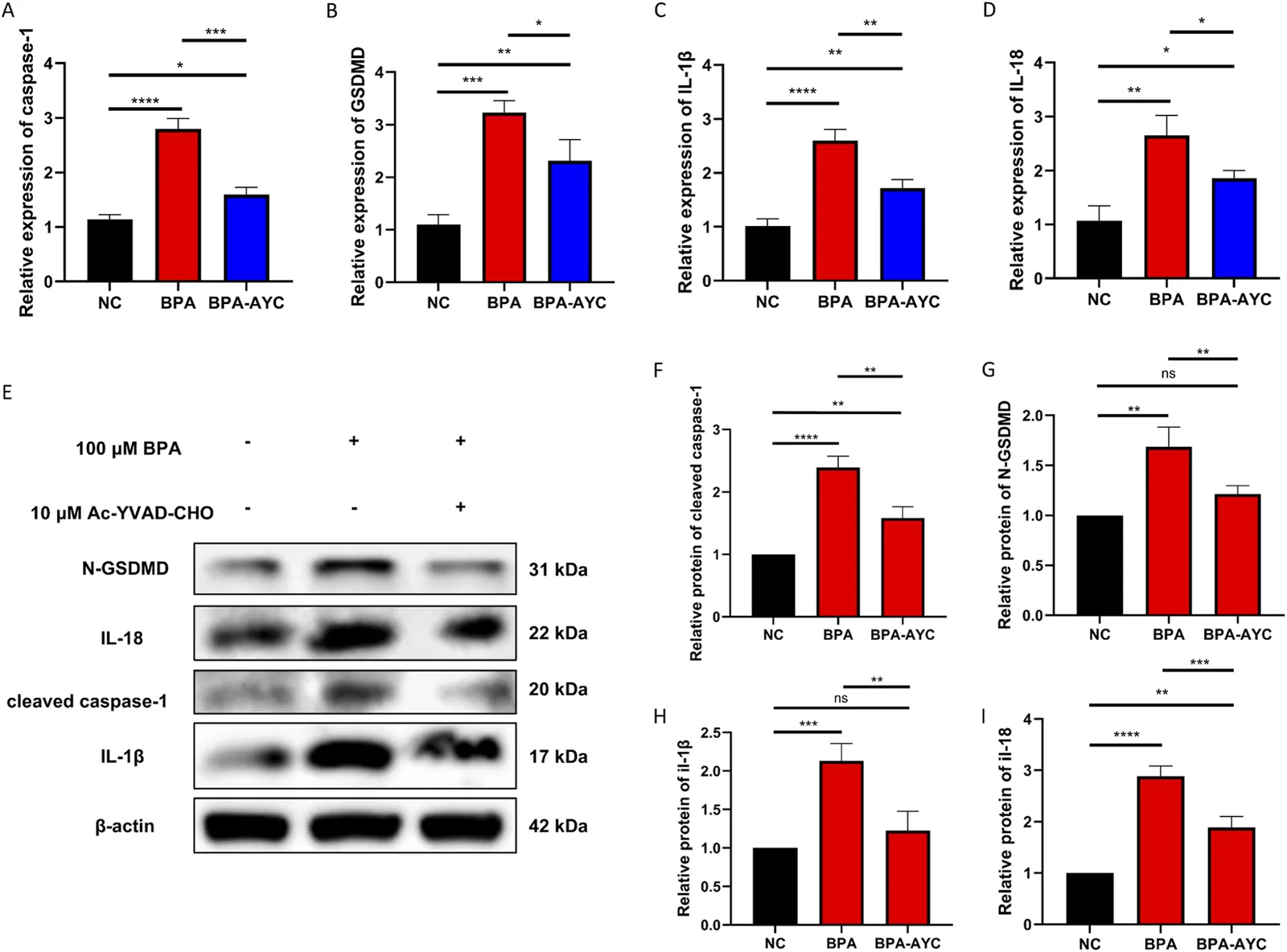
Activation of the CASP1 GSDMD dependent pyroptotic and inflammatory signaling axis by BPA in HCECs. **(A–D)** Relative mRNA expression levels of *caspase-1* **(A)**, *GSDMD* **(B)**, *IL-1*β **(C)**, and *IL-18* **(D)** quantified via qRT PCR analysis. **(E)** Representative Western blot bands illustrating the protein expressions of N GSDMD, IL 18, cleaved caspase 1, IL 1β, and beta actin, with corresponding theoretical molecular weights annotated on the right. **(F–I)** Densitometric evaluation of relative protein levels for cleaved caspase 1 **(F)**, N GSDMD **(G)**, mature IL 1β **(H)**, and mature IL 18 **(I)** normalized against the beta actin internal control. The custom label BPA-AYC denotes human corneal epithelial cells co treated with 100 μM BPA and 10 μM Ac YVAD CHO. All quantitative data are expressed as mean ± SD (n = 3). **Statistical significance is presented via capitalized italicized P values, where *P < 0.05, **P < 0.01, ***P < 0.001, **P < 0.0001, and ns indicates non significance.

To validate the predictions, the mRNA expression of pyroptosis markers and inflammatory cytokines was quantified via qRT-PCR. Compared to the control, 100 μM BPA treatment significantly upregulated the transcription of *caspase-1* (*P* < 0.0001) and *GSDMD* (*P* < 0.001). This was accompanied by increased expression of *IL-1β* (*P* < 0.0001) and *IL-18* (*P* < 0.01). The addition of Ac-YVAD-CHO suppressed the BPA-induced *caspase-1* upregulation and reversed the elevated expression of *GSDMD*, *IL-1β*, and *IL-18* (Figures 14A–D). Western blot analysis was executed (Figure 14E). Densitometric quantification revealed that BPA exposure triggered robust enzymatic cleavage events, evidenced by a substantial accumulation of cleaved caspase 1, mature IL 1β, and mature IL 18 proteins, alongside an increase in the executioner N GSDMD fragment (Figures 14F–I). Co. treatment with the caspase 1 inhibitor significantly suppressed the protein abundance of cleaved caspase 1, mature IL 1β, and mature IL 18. Although the protein level of the N GSDMD fragment exhibited a distinct downward trend upon caspase 1 inhibition, the statistical alteration between the BPA and BPA-AYC groups did not reach absolute significance (Figure 14G). These comprehensive molecular developments demonstrate that BPA drives corneal epithelial cell pyroptosis and downstream inflammatory cytokine maturation through the CASP1 GSDMD dependent signaling cascade.

## 4 Discussion

Dry eye disease (DED) is a multifactorial chronic disorder of the ocular surface. Persistent tear hyperosmolarity directly damages corneal and conjunctival epithelial cells (Stapleton et al., 2025). This damage triggers a local immune-inflammatory cascade and the release of inflammatory mediators, creating a vicious cycle that impairs visual quality (Messmer, 2015). Bisphenol A (BPA) is a classic environmental endocrine disruptor widely used in industrial products like plastics, resulting in high environmental abundance (Adamovsky et al., 2024). Humans ingest BPA primarily through the digestive tract, allowing it to enter the systemic circulation, from which it could hypothetically cross the blood tear barrier to reach the tear film, though definitive empirical validation of this specific transport pathway remains limited. Alternatively, direct environmental deposition represents a highly accessible exposure route; because the ocular surface is directly open to the external environment, corneal epithelial cells undergo continuous physical contact with contaminated indoor dust and settled microparticles. Structurally, BPA closely resembles endogenous estrogens. Its high lipophilicity allows it to easily penetrate biological membranes, including placental, blood brain, and ocular barriers, with the potential to accumulate in lipid rich tissues and cellular organelles (Ighalo et al., 2024). Although BPA has a short *in vivo* half-life of approximately 6 h, large-scale biomonitoring detects its metabolites in the urine of over 90% of tested individuals, indicating a high baseline exposure burden (Radwan et al., 2018). Previous systemic toxicology studies link BPA to oxidative stress and chronic inflammation in multiple organs (Le Corre et al., 2015), yet its specific effects on the ocular surface microenvironment lack mechanistic explanation. By integrating network toxicology, machine learning feature selection, Mendelian randomization, and *in vitro* experiments, this study systematically explored the link between BPA exposure and DED. The results show that BPA damages human corneal epithelial cells by triggering oxidative stress, which activates CASP1-mediated pyroptosis and inflammatory cytokine release.

Toxicity predictions using ADMETlab 3.0 and ProTox 3.0 indicated that BPA possesses significant ocular irritant, corrosive, and *in vitro* cytotoxic properties. Subsequent phenotypic experiments confirmed these predictions. As the first anatomical and immune barrier against environmental stress, the integrity of the corneal epithelium is essential for tolerating ocular surface hyperosmolarity and maintaining tear film dynamics (Baratta et al., 2022). CCK-8, LDH release, and propidium iodide staining assays showed that BPA exposure dose-dependently reduced HCEC viability and disrupted cell membrane integrity. This cytotoxicity corresponds to the microscopic pathological processes of DED, such as the destruction of corneal epithelial microvilli, degradation of intercellular tight junctions, and diffuse epithelial desquamation (Messmer, 2015). These findings provide direct experimental evidence for BPA-induced ocular surface damage.

Functional enrichment analysis indicated that BPA targets were enriched in pro-inflammatory signaling axes, including the NF-κB, TNF, and IL-17 pathways. BPA facilitates abnormal crosstalk between upstream signaling pathways to trigger the NF-κB axis, specifically through TLR4 activation and interference with ERK networks. In the gonads and central nervous system, these molecular events drive oxidative stress and subsequent tissue degeneration (Meng et al., 2020; Wang et al., 2025; Sevastre-Berghian et al., 2022). Beyond direct signaling, BPA remodels the local immune-metabolic microenvironment. This shift promotes the synthesis of IL-17 and TNF-α within adipose tissues and autoimmune-targeted organs (Hong et al., 2023; Dong et al., 2023), and has even been implicated in the inflammatory hyperplasia of the prostate stroma and structural degradation of the spleen (Wang et al., 2022; Xu et al., 2024a). Across diverse physiological contexts, BPA is consistently reported to induce NF-κB, TNF, and IL-17 signaling cascades. NF-κB signaling serves as a central axis in the inflammatory response, and its activation functioning is a primary driver of DED progression. In epithelial cells, hyperosmotic stress and environmental insults trigger NF-κB, directly inducing the release of mediators such as TNF-α and IL-1β (Chu et al., 2024; Zhu et al., 2025; Lin et al., 2025). IL-17 is a key marker of chronic immune injury in DED and synergizes with TNF to disrupt corneal barrier integrity and amplify localized inflammatory cascades (Kim et al., 2025; Garbutcheon-Singh et al., 2019). These mechanisms align with our GO analysis, which highlights oxidative stress and toxicity. Our *in vitro* experiments provided further validation: DCFH-DA staining revealed pronounced ROS accumulation in HCECs following BPA exposure. This redox imbalance causes direct structural damage to the ocular surface barrier and stimulates pro-inflammatory cytokine synthesis, thereby accelerating the pathological process.

Machine learning algorithms identified *CASP1* as the most stable core gene. While the feature selection process also highlighted *TNF*, a detailed comparison justifies our focus on *CASP1.* In terms of topological positions within the interaction network, *TNF* acts as a broad upstream hub associated with systemic immune responses. In contrast, *CASP1* occupies a distinct position tightly linked to the machinery of cellular death. Functionally, *TNF* regulates general leukocyte recruitment and cytokine cascades. Meanwhile, *CASP1* specifically executes pyroptosis by cleaving gasdermin D. This cleavage directly leads to physical cell membrane rupture and corneal epithelial barrier dysfunction. Regarding druggability, current *TNF* inhibitors are primarily systemic biologics. These agents carry risks of profound immunosuppression and are difficult to formulate for stable ocular delivery. On the other hand, *CASP1* represents an intracellular enzymatic target. This characteristic makes it highly suitable for the development of small molecule inhibitors designed for topical ophthalmic application. Therefore, *CASP1* offers a more precise and practical target for localized intervention in DED. Mendelian randomization demonstrated that increased CASP1 expression causally elevates DED risk (OR = 1.11), with no indication of heterogeneity or horizontal pleiotropy. Machine learning and Mendelian randomization suggest that CASP1 is a key component in the pathology and susceptibility of DED. And the connection between the toxicant and CASP1 activation is confirmed by our laboratory experiments. The human *CASP1* gene, located at chromosome 11q22, encodes the inflammatory protease caspase-1 (Zhen et al., 2024; Sollberger et al., 2014). Unlike apoptotic caspases, caspase-1 uses its N-terminal CARD domain to assemble into the inflammasome (He et al., 2016). Caspase-1 has been proposed as a sensitive biomarker for ocular surface damage (Tovar et al., 2022). Evidence has positioned the NLRP3/caspase-1/GSDMD-mediated pyroptotic pathway as a significant contributor to ocular surface dysfunction. Hyperosmotic stress upregulates caspase-1 activity, driving HCEC pyroptosis and compromising the corneal epithelial barrier (Li et al., 2022). Targeting this axis has been shown to alleviate corneal epithelial injury in models induced by benzalkonium chloride (BAC) or hyperosmotic stress (Lou et al., 2023; Zhang J. et al., 2021). In the BAC-induced model, the accumulation of reactive oxygen species (ROS) and subsequent oxidation of mitochondrial DNA (mt-DNA) appear to be the triggers for NLRP3 inflammasome activation (Lou et al., 2023). Moreover, caspase-1-dependent pyroptosis contributes to the pathogenesis of meibomian gland dysfunction (Wu et al., 2025). ROS accumulation serves as a classic upstream trigger for inflammasome assembly (Pandey et al., 2025). Activation of the inflammasome triggers caspase-1 to cleave its downstream substrate GSDMD, leading to membrane pore formation, cytosolic leakage (pyroptosis), and the maturation of pro-inflammatory cytokines IL-1β and IL-18 (Pandey et al., 2025; Xu and Núñez, 2023; Shi et al., 2017; Yu et al., 2021). This cascade not only executes programmed cell death in corneal epithelial cells but also amplifies local inflammatory responses, directly compromising ocular surface barrier integrity and tear film stability (Zhang Y. et al., 2021). Systemic toxicological models mirror this mechanism: BPA directly induces osteocyte pyroptosis via the ROS/NLRP3/caspase-1 axis (Zhang et al., 2022), hyperactivates inflammasomes in myeloid cells (Panchanathan et al., 2015), and exacerbates liver metabolic dysfunction through oxidative crosstalk (Pirozzi et al., 2020). We hypothesized that BPA exacerbates DED by upregulating *CASP1* and hyperactivating the ocular surface inflammasome-pyroptosis axis. Our qRT-PCR and Western blot analyses demonstrated that BPA exposure markedly elevated the expression of caspase-1, GSDMD, and downstream cytokines in HCECs at both the mRNA and active protein levels. The application of the caspase-1 inhibitor Ac-YVAD-CHO successfully reversed these transcriptomic alterations, mitigated ROS accumulation, and significantly reduced the protein abundances of cleaved caspase-1, mature IL-1β, and mature IL-18. Intriguingly, although the decrease in N-GSDMD protein levels did not achieve absolute statistical significance upon caspase-1 impedance, the comprehensive attenuation of downstream mature cytokines and the robust rescue of cell survival strongly validate that blocking this molecular junction remains functionally effective. These validations confirm that BPA mediates cellular pyroptosis and exacerbates ocular surface inflammation through the activation of the CASP1/GSDMD axis.

GSEA revealed a strong correlation between elevated *CASP1* expression and enhanced mitochondrial oxidative phosphorylation (OXPHOS) alongside increased ribosomal function. Upregulated OXPHOS exacerbates electron transport chain leakage, generating a continuous ROS supply that serves as a persistent trigger for NLRP3 inflammasome assembly (Kelley et al., 2019). Heightened ribosomal activity supplies the essential biosynthetic foundation for translating pro-inflammatory mediators. Such metabolic vigorousness is highly consistent with the energetic requirements of caspase-1 as an inflammatory effector enzyme. A corresponding negative enrichment in transcriptional regulation pathways confirms this cellular transition toward the execution of inflammation.

When evaluating immune infiltration, we observed a positive correlation between *CASP1* expression levels and M0 macrophage abundance in the aggregated data. However, stratifying the samples by disease status significantly attenuated this association, demonstrating Simpson’s paradox, a statistical phenomenon where a global trend disappears within specific subgroups (Ameringer et al., 2009). This statistical discrepancy confirms that the observed association is primarily driven by the overall disease status rather than direct regulatory relationships. Within the ocular surface microenvironment, CASP1 primarily functions as an intracellular inflammatory effector that processes downstream cytokines rather than a direct driver of immune cell recruitment. Toxicology studies must assess functional cellular activation states rather than relying solely on simple cell counts.

Overall, the present study integrates *in silico* toxicological predictions, Mendelian randomization, and *in vitro* validation to explore the BPA-DED axis. Despite multiple lines of evidence, objective limitations remain. The screening strategy relied on intersecting predicted targets of BPA with differentially expressed genes from corneal tissues of patients with DED. These altered genes may reflect the consequences of advanced disease stages. Therefore, this approach cannot fully differentiate between biological causes and consequences. Future validation requires the use of tissue specific data or transcriptomic profiles obtained under direct exposure to BPA. The integration of transcriptomic datasets with small sample sizes represents another limitation. Although the batch correction protocol successfully aligned the mean expression vectors, variance differences remained. The concentration ellipses exhibited distinct orientations, which reflects the statistical instability of small sample groups containing only four or five replicates. This variance heterogeneity represents study specific characteristics that persist after mean adjustment, which may introduce a selection bias during the initial differential gene screening. Future investigations using larger datasets are required to minimize these selection biases. The instrumental variables for CASP1 expression were derived from whole blood eQTL data due to the lack of ocular tissue specific databases. Although blood serves as a proxy for systemic inflammatory pathways, this choice introduces a potential tissue specific bias. Future validation requires corneal tissue specific genetic association datasets to confirm these local regulatory effects. Another limitation involves the high concentration of BPA used for our cellular validation. The concentration of 100 μM is higher than typical human environmental exposure levels. The primary purpose of this study is not to represent daily human contact, but rather to discover and characterize the downstream pathological processes and molecular mechanisms driven by BPA exposure. Specifically, micromolar concentrations of BPA have been shown to induce DNA damage and apoptosis in human epithelial cells (George and Rupasinghe, 2018), activate the ROS and caspase 1 pyroptotic axis in immune cell models (Xu et al., 2024b), and trigger robust para inflammatory responses in *in vitro* epithelial models (Loffredo et al., 2020). However, these acute parameters do not completely represent the chronic accumulation patterns found in real world settings. Future investigations must include experiments using lower concentrations over longer exposure periods to better simulate human environmental conditions. A single cell line model cannot emulate the complex microenvironment of the ocular surface, missing the interactions among the corneal epithelium, conjunctival goblet cells, and immune populations. Future research requires confirmation in animal models and clinical samples to validate these mechanisms. Additional laboratory studies are also necessary to strengthen our observations. These investigations should include the assessment of genetic knockdown using small interfering RNA and the evaluation of alternative specific inhibitors. Developing topical ophthalmic formulations that block the CASP1/GSDMD axis offers a strategy for managing DED.

## 5 Conclusion

This study identifies CASP1 as the central molecular hub linking BPA exposure to DED. BPA exposure impairs HCEC viability and triggers membrane damage alongside a surge in intracellular ROS. BPA promotes ocular surface injury by activating the CASP1/GSDMD-mediated pyroptotic axis and its associated inflammatory cascade. Inhibition of CASP1 effectively abrogates this process, suggesting potential avenues for clinical intervention.

## Data Availability

The raw data supporting the conclusions of this article will be made available by the authors, without undue reservation.
